# Unique adjustable UC luminescence pattern and directional radiation of peculiar-shaped NaYF_4_: Yb^3+^/Er^3+^ microcrystal particle

**DOI:** 10.1038/s41598-017-04519-6

**Published:** 2017-07-14

**Authors:** Qingyan Han, Chengyun Zhang, Chi Wang, Zhaojin Wang, Caixia Li, Wei Gao, Jun Dong, Enjie He, Zhenglong Zhang, Hairong Zheng

**Affiliations:** School of Physics and Information Technology, Shaanxi Normal University, 710119 Xi’an, China

## Abstract

A new design of peculiar-shaped β-NaYF_4_: 20% Yb^3+^/2% Er^3+^ hexagonal microcrystal (PSβHM) is proposed and its upconversion (UC) luminescence with adjustable color pattern is studied with near infrared excitation. Flower-like green UC luminescence emission pattern from blooming to withering process and intensive directional red emission are achieved by simply adjusting the focal point position of the excitation light. The mechanism that determines the unique UC luminescence phenomena are investigated systematically. The function of the internal light reflection and waveguide effect with annular microcavity is explored based on the structure characteristic of the hexagonal microplate. The current work may have great significant and potential applications in the development of optoelectronic device, color display, directional light and laser emission of microsystem.

## Introduction

Lanthanide-doped upconversion (UC) is a multiphoton processes that is characterized by simultaneous or successive absorption of two or more low-energy photons via intermediate energy states, followed by a high-energy photon emission^1^. Long luminescence lifetime and narrow emission bandwidth are characteristic features of UC luminescence emission due to parity-forbidden principle of f-f transition^2, 3^. Unique optical properties including high signal to noise ratio, large anti-stokes shift, high photochemical stability and low toxicity, are typical for lanthanide doped luminescence materials^4, 5^. These characteristics make lanthanide-doped UC luminescence materials very attractive in fundamental research and practical oriented applications including solar cell^6^, biological imaging^7^, 3D display^8^, optical storage^9^ and photo switching^10^. Among lanthanide-doped UC materials, fluoride is well known as a prominent matrix due to its low energy phonons and high chemical stability^11^. As one of the most effective UC host materials, hexagonal NaYF_4_ has very low maximal optical phonon energy that suppresses nonradiative multiphonon relaxation effectively^12^. Many reports on the investigation of material preparation and UC luminescence emission have been presented, but directional UC luminescence emission and emission with distinguished patterns are rarely seen for single UC luminescence particle^13, 14^.

Luminescence waveguide based on the assembly of organic molecules, polymers and semiconductors, has been built to produce guided light under ultraviolet excitation^15–19^. Compared with these, the waveguide based on the UC luminescence emission has been presented as a promising alternative to produce directional luminescence emission^14^. Since near-infrared light is more penetrative and less destructive, the UC luminescence waveguide favors the potential application in optical communication^20^ and biology images^21^. Light emission produced by dopant lanthanide could propagate along certain direction of the host material, therefore, directional UC emission should be obtained with fixed color gamut and pattern by controlling the composition of lanthanide dopant in microcrystal, the configuration of the particle morphology as well as the excitation condition^22^.

In current work, we present a novel UC emission of peculiar-shaped β-NaYF_4_: 20% Yb^3+^/2% Er^3+^ single microcrystal (PSβHM) with unique morphology. Bright UC emission with unique and attractive patterns are obtained with near infrared (NIR) excitation. A beautiful flower-like green UC emission﻿ pattern﻿ from blooming to withering process is presented continuously through simply tuning the focal point position of the excitation light. Directional red light UC emission is also successfully obtained by properly selecting the excitation position. The possible mechanism and UC luminescence from multiple connected PSβHM is proposed.

## Materials and Methods

### Materials

Y(NO_3_)_3_ · 6H_2_O, Yb(NO_3_)_3_ · 6H_2_O and Er(NO_3_)_3_ · 6H_2_O are obtained by dissolving Y_2_O_3_, Yb_2_O_3_ and Er_2_O_3_ (99.99%. Sigma-Aldrich) in dilute nitric acid, respectively. During the preparation process, the solution is stirred at 60 °C for several hours to evaporate the superfluous nitric acid. Then, it was dissolved in deionized water to form RE(NO_3_)_3_ solution. Sodium Fluoride (NaF, 98.00%), and sodium citrate (Na_3_C_6_H_5_O_7_ · 2H_2_O, 99.00%) with analytic grade were supplied by Sinopharm Chemical Reagent Co., Ltd. (China). Deionized water was used throughout the experiment. Unless otherwise stated, all the chemicals were used without further purification.

### Materials synthesis

High quality peculiar-shaped β-NaYF_4_: 20% Yb^3+^/2% Er^3+^ hexagonal microcrystals (PSβHMs) were synthesized via modified hydrothermal method with assistance of sodium citrate to control the crystal growth^23, 24^. In a typical process, 0.5 mmol sodium citrate was first dissolved in 21.0 ml of deionized water, and then a mixture containing 0.3 mmol aqueous solution of RE(NO_3_)_3_ (RE = Y, Yb, Er; Y^3+^/Yb^3+^/Er^3+^ = 39/10/1) was added. The solution was kept stirring for 30 min. Thereafter, the aqueous solution of NaF (1.0 M; 6.0 ml) was added to form the mixture with vigorous stirring for about 25 min. Subsequently, the mixture was transferred into 50 ml Teflon-lined autoclave and heated at 200 °C for 24 hours. After cooling down to room temperature, the final product was collected by centrifugation, washed with deionized water and ethanol repeatedly. The finally samples were dried at 60 °C for 12 hours.

### Characterization methods

Measurement with scanning electron microscope (SEM, FEI- Nova NanoSEM 450) was conducted by operating at 10 kV for characterizing the sample morphology. High-resolution transmission electron microscopy (HRTEM) analyses was carried out with FEI-Tecnai G2 F20 Field-emission transmission electron microscope (FE-TEM) operating at an acceleration voltage of 200 kV. The powder X-ray diffraction (XRD) measurement was done on a Rigaku D/Max2550VB+/PC diffractometer at a scanning rate of 4° min^−1^ with graphite monochromatic Cu K*a* (40 kV, 40 mA) radiation (λ = 0.154 06 nm). UC luminescence emission was obtained with a Quanta Ray Lab-170 YAG: Nd^3+^ pulse laser (SHG: 532 nm, Spectra Physics) and a Ti sapphire femtosecond laser (Mira 900-F, Coherent) as an excitation source. A spectrometer (SP2750i, 0.008 nm) equipped with PIXIS 100 charge coupled device (CCD, ACTON) and PD471 photomultiplier tube (PMT, ACTON) were used for luminescence detection. The luminescence photographs were obtained through the confocal microscopy (OLYMPUS-BX51) equipped with camera Canon 75 600D. All of the measurements were conducted at room temperature.

## Results and Discussion

**Figure 1:**
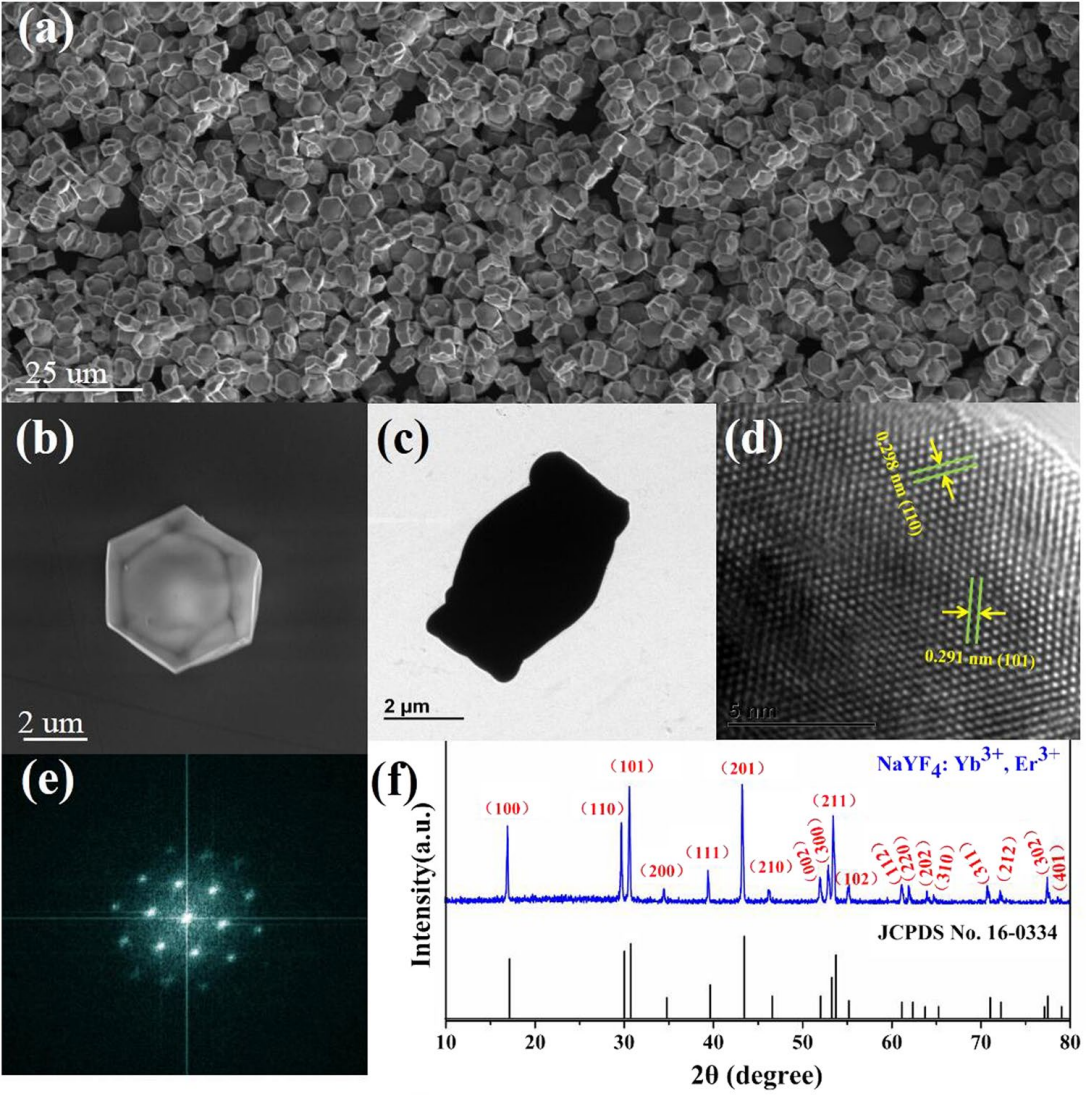
(**a**) SEM image of the as-synthesized peculiar-shaped NaYF_4_: 20% Yb^3+^/2% Er^3+^ hexagonal microcrystals. (**b**) SEM image of the single microcrystal. (**c**) TEM image of the single microcrystal. (**d**) HRTEM image of the microcrystal. (**e**) Corresponding Fourier transform diffraction pattern of the HRTEM. (**f**) XRD pattern of the microcrystal and literature data for hexagonal phase NaYF_4_ (JCPDS file number 16–0334).

The morphology and crystal structure of the sample particle are characterized by conducting SEM, HRTEM, and XRD measurements, as shown in Fig. 1. According to the SEM image presented in Fig. 1(a) and (b), uniformly distributed microcrystals are arc-shaped hexagonal plates with side walls around the outside edges. The average overall dimension of each plate is about 2.0 µm in thick and 5.0 µm in diameter. Six petal-shaped side walls have the side length of about 3.4 µm and height of about 2.5 µm. The HRTEM image taken from a corner of the PSβHM is presented in Fig. 1(c). Figure 1(d) shows the lattice fringe with observed interplanar spacing of 0.298 nm and 0.291 nm, which corresponds to lattice space in (110) and (101) planes of β-NaYF_4_, respectively, revealing the high crystalline nature of the particle. The electron diffraction pattern obtained from the Fourier transform of HRTEM image also confirms the single-crystalline tetragonal phase of the microcrystal sample, which is shown in the Fig. 1(e). In the XRD spectrum shown in Fig. 1(f), all of diffraction peaks are well indexed in accordance with β-NaYF_4_ crystal (JCPDS file NO. 16–0334), indicating the tetragonal phase of the microcrystals with high crystallinity and purity.

**Figure 2:**
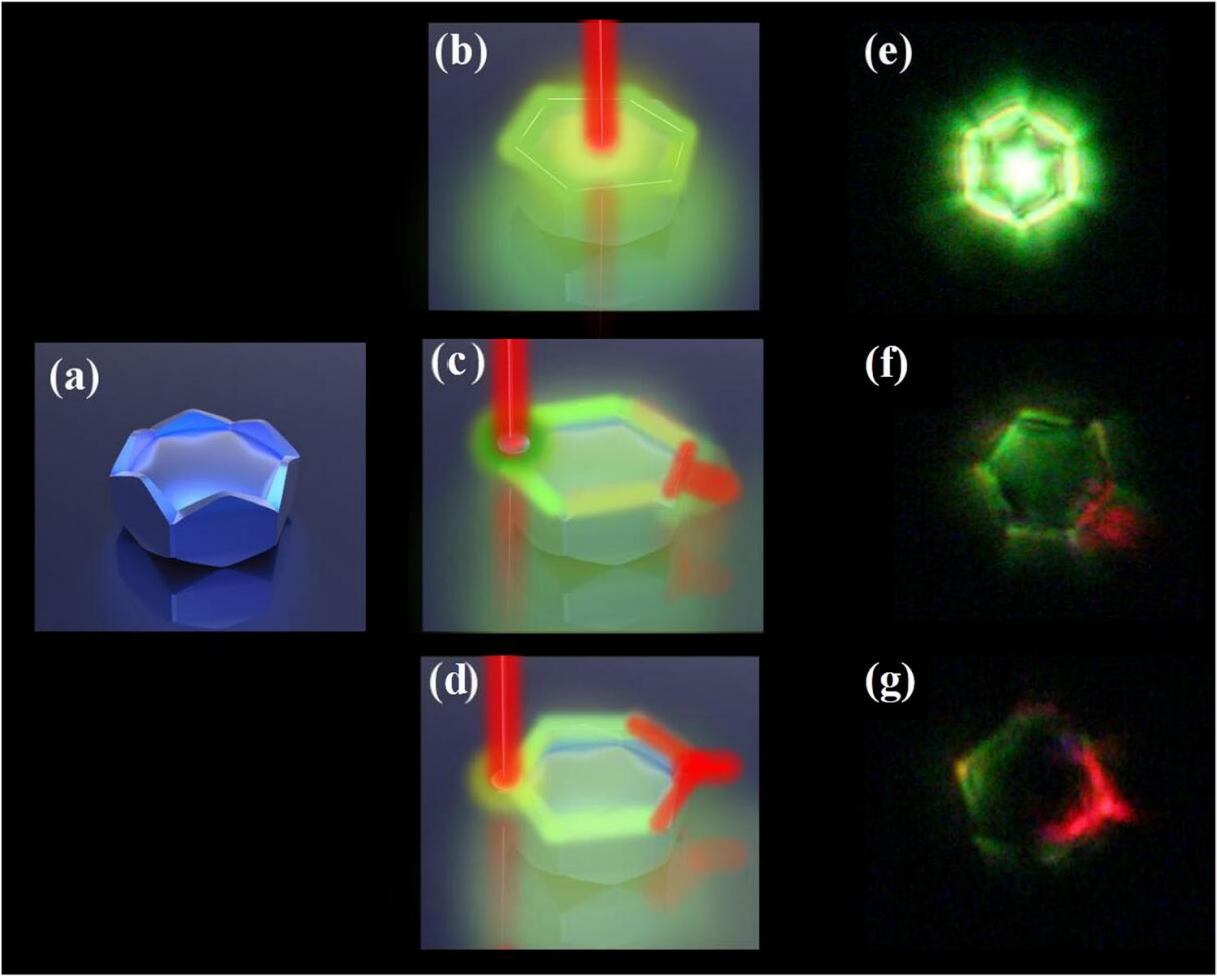
(**a**) Schematic illustration of the microcrystal structure. (**b**) to (**d**) Schematic illustration of PSβHM under specific location of the excitation light. (**e**) to (**g**) Typical UC luminescence emission observed with corresponding location of the excitation light.

The property of UC luminescence emission from a single PSβHM is performed by a confocal setup equipped with an optical microscope (OLYMPUS-BX51). Each single microcrystal is excited by a focused 980 nm continuous wave (CW) laser. The specific focal point location of the excitation light is adjusted by fine movement of the microcrystal particle on the quartz substrate that is mounted on a 3D moveable stage. The UC luminescence spectra and emission patterns from the single microcrystal are investigated carefully under different excitation condition. Figure 2 presents the schematic structure and the unique adjustable UC luminescence properties of the single PSβHM particle, in which the center, side edge, and the corner of the particle are excited with different depth of focal point in the sample.

**Figure 3:**
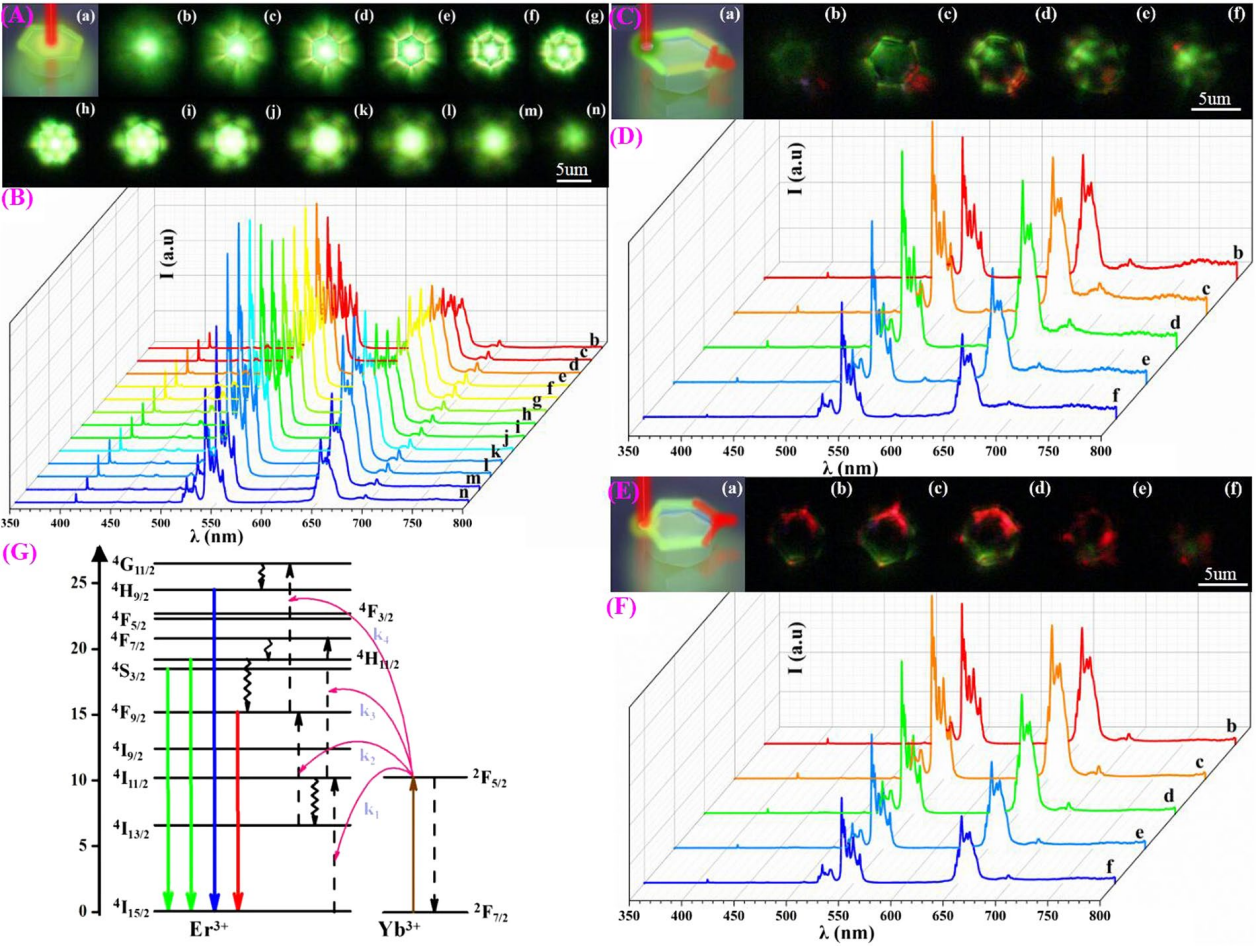
UC emission spectra and luminescence pattern photographs from single PSβHM on a quartz substrate. (**A**) and (**B**), (**C**) and (**D**), (**E**) and (**F**) are luminescence emission patterns and spectra when the particle is excited at the center, side edge, and corner of the microcrystal particle, respectively. (b) to (n) in the Figures (**A**) and (**B**), (b) to (f) in the Figures (**C**) and (**D**), (**E**) and (**F**) correspond to different vertical position of the excitation focal point (During the experimental measurement, the depth of the excitation focal point in each case is gradually moved for the upper to the bottom surface of the particle by fine-tuning the vertical position of the sample.). All (a) of the Figures (**A**), (**C**), and (**E**) present the schematic illustration of the sample and the specific location of the excitation light. Figure (**G**) is the schematic illustration of the energy level and corresponding transitions.

Figure 3(A) to (F) show the results of experimental observation for the three different excitation positions of a single PSβHM. As it is shown in Fig. 3(A), flower-like green UC luminescence emission pattern is exhibited when NIR excitation light is focused on the central of the microcrystal particle. A fascinating phenomenon appears and the emission pattern changes accordingly as the depth of the focal point in the sample changes gradually. The luminescence emission pattern changes like a flower from blooming to withering with the depth change of the excitation focal point. We have to point out that the emission patterns recorded by optical camera present in bright green, while the result of the spectral measurement shown in Fig. 3(B) indicates the observation of green and red emission at 540 and 654 nm, for which the green emission is stronger than the red one. When the excitation laser beam is focused on one side of the hexagonal microcrystal particle and moves gradually from the top to the bottom surface, very different phenomenon is detected (Fig. 3(C)). The beautiful flower-like bright green patterns are fading away comparing with the case presented in Fig. 3(A), and bright directional red emission mainly appears at only one side of the hexagonal microcrystal particle. The position of the directional red emission is always on the symmetry axis through the excitation point and the center of hexagonal particle. The brightest directional red luminescence launches when the depth of the focal point is adjusted vertically to the mid position. Meanwhile, the luminescence spectral measurement reveals the similar spectral property with that presented by Fig. 3(B). When the focused excitation laser beam is moved to the corner of hexagonal microcrystal particle (Fig. 3(E)), the green emission pattern seeing in the Fig. 3(A) is very hard to be observed in this case, while very bright and directional red luminescence emission is exhibited from the corner of the hexagonal microcrystal particle, for which the position is exactly on the central symmetry axis passing through the excitation point. We also noticed that the light mainly emits radially from the six outer sides of the hexagonal microcrystal particle for the case of Fig. 3(A), while the light propagates mainly along the outside circle and the red luminescence launches out the sample directionally from one side or corner of the particle for the cases of Fig. 3(C) and (E). The overlap of white light image with the fluorescence one of PSβHM and UC luminescence for the single peculiar-shaped β-NaYF_4_: 20% Yb^3+^/2% Tm^3+^ hexagonal microcrystal were further verified these phenomena in the Supporting Information Figures S1 and S2. The luminescence spectral measurements present similar spectral features as that presented in Fig. 3(B),(D) and (F). In all of three cases described above, the luminescence intensity changes with vertical movement of the excitation focal point in the sample. It is found that when the excitation laser beam is moved to the center of the particle and changes gradually as the depth of the focal point from the top to the bottom surface, the corresponding R/G ratio is less than one. But when the excitation laser beam is focused on the side or corner of the hexagonal microcrystal particle and moves from the top to the bottom surface, the corresponding R/G ratios are greater than one (Supporting Information Figure S3). However, the brightest emission corresponds to the situation when the focal point is at the mid of the vertical position. Therefore, when the single PSβHM is excited at the central, side, and corner of the particle, bright flower-like green and directional red luminescence emissions are obtained from the single particle by simply adjusting the excitation focal point position. As shown in the Fig. 3(G), characteristic emission bands in spectra are mainly at 654 nm, 540 nm and 520 nm, which correspond to transitions of ^4^F_9/2_ → ^4^I_15/2_, ^4^S_3/2_ → ^4^I_15/2_ and ^2^H_11/2_ → ^4^I_15/2_ of Er^3+^ in the particle, respectively^25^. Very weak emission at 411 nm is resulted from the transition of ^2^H_9/2_ → ^4^I_15/2_. It should be mentioned that the observation of 411 nm weak blue emission reveals the high luminescence efficiency of PSβHM since it is generally very hard to be detected due to the very low efficiency of three-photon UC process^26^. Furthermore, to well prove the mechanism of the population for ^4^S_3/2_ and ^4^F_9/2_ states under NIR excitation, the pumping power dependence of the green and red emission intensity is separately investigated for the three different excitation positions of PSβHM (Supporting Information Figures S4 and 5). With decrease of the pump power, the luminescence intensity decreases gradually for both the green and the red emissions in the three cases. Interesting, when pump power density was decreased, the directional red emissions are more and more obvious under excitation position at the side and corner of the particle.

The phenomena presented in Fig. 3 could be explained with total internal reflection and optical waveguide effect. When the excitation laser is focused on the center of the hexagonal microcrystal particle, a regular luminescence emission pattern presents. The luminescence emission excited by NIR light could be regarded as a point light source emitting in all directions randomly. When the ions near the top or bottom surface of the particle are excited, the UC luminescence radiation does not propagate inside the particle, light scattering should be the main process for this case. As the focal point of the excitation light moves vertically towards the central area of the particle, beautiful flower-like UC luminescence emission pattern forms. The pattern fades out gradually as the excitation focal point moves away from the central position, and the intensity ratio of red to green emission varies accordingly. In this case, the PSβHM acts as a luminescence-based waveguide transmitting UC luminescence toward the six sides radially. Considering the unique structure of the particle and the specific refractive index of the host material, the observed luminescence pattern should be mainly resulted from total internal reflection effect. The guided UC luminescence is scattered and launches out the sample from six sides of the microcrystal particle. Through total internal reflection and light scattering effect, the NIR excitation light and UC luminescence transmit radially to the side edge of the particle that re-excites and accumulates the UC luminescence emission at six edge sides forming the observed flower-like pattern. Since the light scattering of the green emission is stronger than the red one, only obvious green luminescence pattern is recorded with camera though the red emission also exists (Supporting Information Figure S6).

When the excitation light is focused on the outside edge of the hexagonal microcrystal particle, the NIR excitation light and UC emission mainly transmit along the edge lace of the microcrystal particle through total internal reflection. The special configuration of the hexagonal microcrystal, especially its side lace presents characteristic resonant microcavity for luminescence propagation and serves as a light waveguide around the particle. During the propagation process, the green light decreases gradually and emits out from every side of the hexagonal microcrystal. While the directional red emission light keep transmitting along the side lace and launches out of the particle from the side that is on the symmetry axis passing through the excitation point and the center of the hexagonal microcrystal. As the focal point of the excitation light moves vertically inside the particle, the intensity of the directional red UC luminescence emission changes accordingly. The strongest directional red luminescence emission occurs when the excitation point is at the middle position. However, it decreases gradually as the excitation focal point moves away from the middle of the vertical position. In this case, the UC luminescence is guided actively through incessantly repeated re-absorption and re-emitting processes along the propagation direction, resulting in re-distribution of the guided light from short to long wavelength with increasing propagation distance. The re-absorption and re-emitting processes could increase the population of ^4^F_9/2_ state of Er^3+^ leading to the enhanced red and reduced green emission. As it is presented UC mechanism for the single PSβHM in the Fig. 3(G), the Yb^3+^ ions absorb NIR light photon energy and then transfer energy to Er^3+^. However, as a result of the unique structure of the PSβHM, the PSβHM could act as a luminescence-based waveguide transmitting the NIR excitation light and UC luminescence along their edge lace through total internal reflection, which increases the optical path of the excitation light. It leads to enhance the excited-state absorption via Yb^3+^ ions during the propagation process. Therefore, the red UC emission resulting from the transition of the excited ^4^F_9/2_ to ^4^I_15/2_ ground state is enhanced.

When the excitation laser beam is focused on the corner of the hexagonal microcrystal particle, the observed phenomenon is similar to what happened when the excitation light is at the side of the hexagonal plate. The UC emission also propagates along the side lace of the microcrystal and directional red emission exhibits at the symmetry corner, but the green light is rarely seen in this case. Similar to the discussion we have just made above, the phenomenon is also own to the unique structure of the PSβHM that acts as an annular resonant microcavity, which increases the excited-state absorption via Yb^3+^ ions and enhances the population of red emitting state ^4^F_9/2_.

Optical waveguide effect is due to total reflection effect that results from the refractive index difference. In this case, the medium that transmits the light has a larger refractive index than that of the surrounding dielectric environment, leading to the light confinement in the medium. According to Snell’s law *n*_1_ sin *θ*_1_ = *n*_2_ sin *θ*_2_, the critical angle for the total internal reflection of NaYF_4_ is *θ*_2_ = *θ*_*c*_ = 44.4° when *θ*_1_ = 90°. Here the refractive index of NaYF_4_ is *n*_2_ = 1.43 and that of the air is *n*_1_ = 1.00^27^. Oppositely, the refractive index of the surrounding dielectric environment is higher than the sample matrix medium, total reflection and waveguide effect should not occur, and the phenomena presented in the Fig. 3 could not be observed.

**Figure 4:**
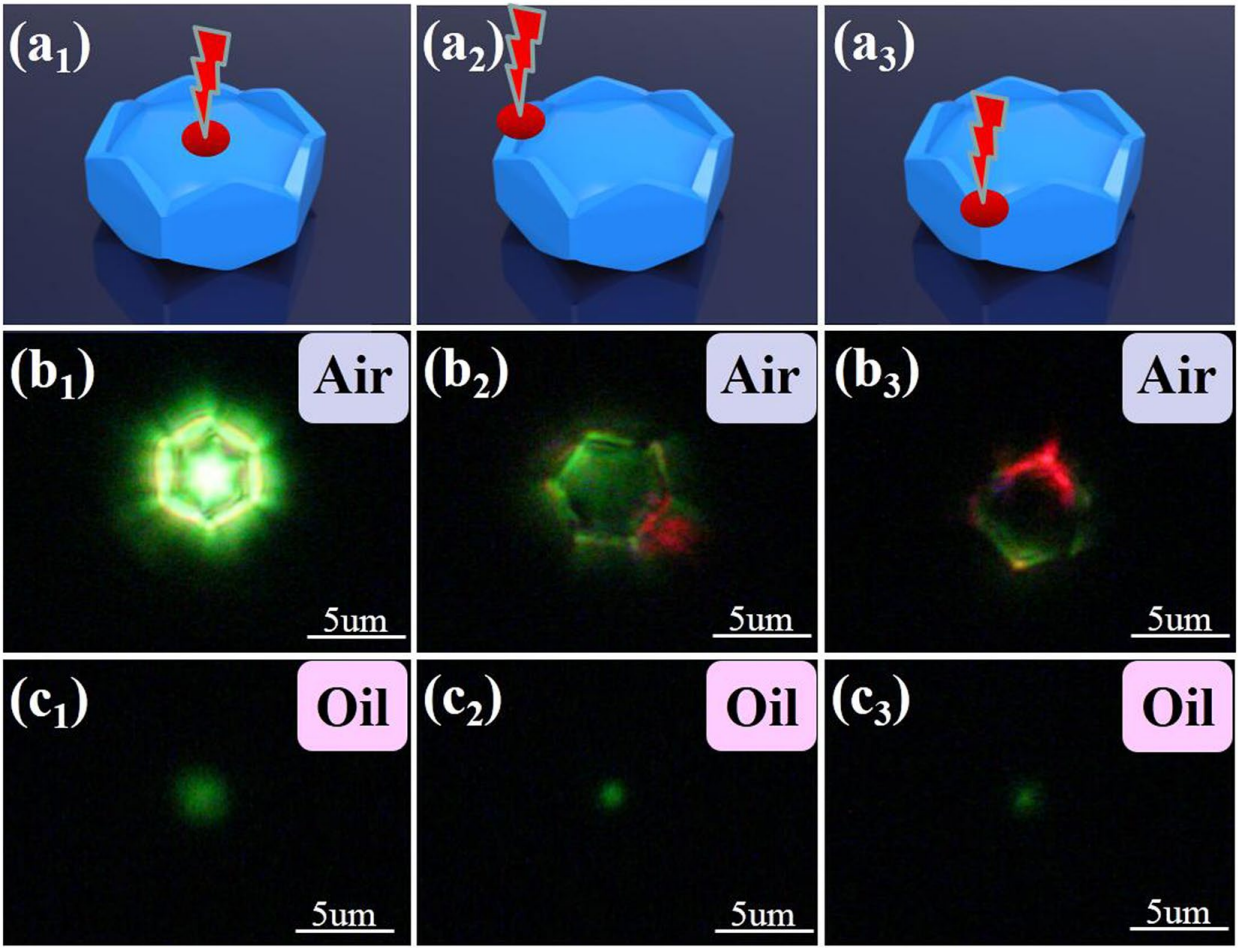
Sample illustration and UC luminescence emission of PSβHM dispersed in different surrounding medium. (**a**_**1**_) to (**a**_**3**_) Specific location of the excitation light. (**b**_**1**_) to (**b**_**3**_) Typical UC luminescence emission observed with corresponding location of the excitation light when the sample particle is in the air. (**c**_**1**_) to (**c**_**3**_) Typical UC luminescence emission observed with corresponding location of the excitation light when the sample particle is in the microscopy oil. The refractive index of air, microscopy oil and NaYF_4_ medium are 1.00, 1.59 and 1.43, respectively.

To support the above discussion on the PSβHM, we have also conducted the experimental study on the regular hexagonal flat microcrystal plate with similar dimension, and the observation of PSβHM in the higher refractive index medium such as microscopy oil. Compared with what presented by PSβHM, the conventional hexagonal microcrystal did not present any flower-like green luminescence pattern or directional red emission (Supporting Information Figure S7). This suggests that the unique structure of the PSβHMs does provide a specific situation for the effect of total reflection and light waveguide. When we immersed the PSβHM in microscopy oil, for which the refractive index (*n* = 1.59) is higher than the matrix medium NaYF_4_ (*n*_2_ = 1.43), flower-like pattern and directional red emission could not be detected (Fig. 4(c1) to (c3)). These results confirm the proposed mechanism on the formation of UC emissions (Figs 3 and 4(b1–b3)). All of the discussion indicates that the PSβHM can act as a waveguide modulator, which may have significant potential application in wavelength-sensitive optical device and color display.

**Figure 5:**
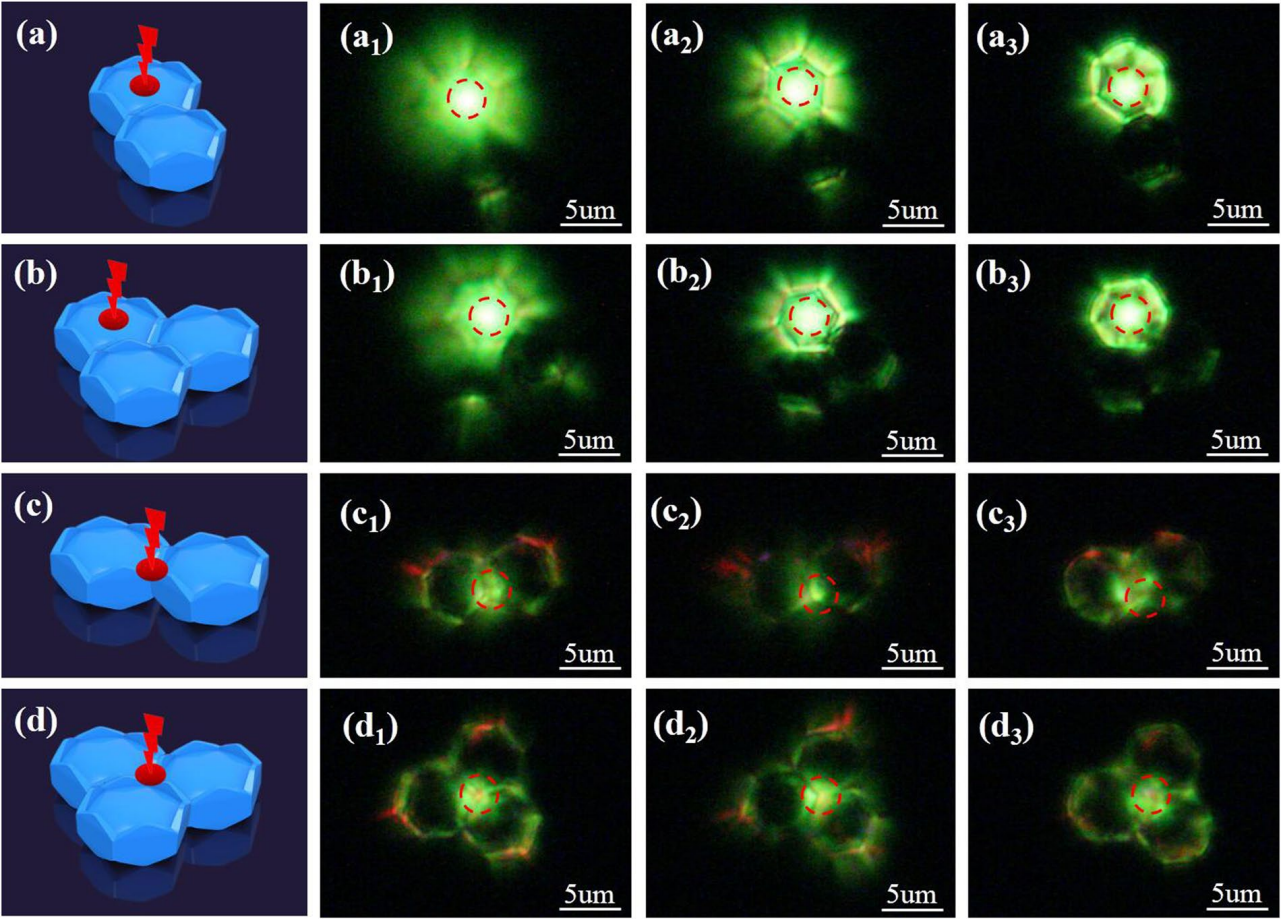
Sample illustration and photographs of UC luminescence from closely situated PSβHMs. (**a**), (**b**), (**c**) and (**d**) are schematic illustration of the sample configuration and the position of the excitation light. (a_1–3_) and (c_1–3_) are emissions of dimer microcrystals that are excited at the center and the corner of one particle from the top to the middle surface, respectively. (b_1–3_) and (d_1–3_) are emissions of trimer microcrystals that are excited at the center and the corner of three neighbored hexagonal microcrystals from the top to the middle surface, respectively. Red dash-line circles indicate the specific position of the excitation light in the experimental study.

Further investigation on the UC luminescence from multi-PSβHMs also shows the light waveguide effect of the sample. As it is presented in Fig. 5, in the system of dimer or trimer PSβHMs, the bright UC green luminescence not only emits from the particle whose center is directly excited, but also emits from the one that is not directly excited by NIR light. This result implies that the emission light can also propagates along the sides from one microcrystal particle to its neighbored one through the connecting edge or point. Furthermore, directional red UC emission also launches out of the system from all sides or corners that are on the axis line connecting the excitation point and the center of the hexagonal microcrystal particles. The emission light propagates from one microcrystal particle to the other through the connecting points or side edges, light is easily coupled and transmitted among neighbored microcrystal particles. These phenomena support that the UC light propagates along the annular resonant microcavity in the PSβHM formed naturally by sample itself.

## Conclusions

A newly designed PSβHM is synthesized and unique UC emissions with adjustable flower-like pattern and directional radiation are reported for the first time. The characteristic morphology of the microcrystal provides a necessary condition for the observation of the very attractive UC luminescence. The phenomena of green luminescence flower from blooming to withering and directional red emission are presented by simply adjusting the focal point position of the excitation light. It is suggested that the internal reflection and light waveguide effect determine the typical feature of the UC emission from the microcrystal particle. The current design and study should have great significance in the development of micro-optoelectronic devices, pattern-changeable three-dimensional color display, and directional light emission and micro-laser systems.

## Electronic supplementary material

Unique adjustable UC luminescence pattern and directional radiation of peculiar-shaped NaYF4:Yb3+/Er3+ microcrystal particle
