# Supplementary material for: Unique adjustable UC luminescence pattern and directional radiation of peculiar-shaped NaYF4: Yb3+/Er3+ microcrystal particle
Source: Sci Rep. 2017 Jul 14;7:5371. doi: 10.1038/s41598-017-04519-6 (PMC5511274; doi:10.1038/s41598-017-04519-6)
Supplement: Supplementary file 1 — Unique adjustable UC luminescence pattern and directional radiation of peculiar-shaped NaYF4:Yb3+/Er3+ microcrystal particle [file 41598_2017_4519_MOESM1_ESM.pdf]

Unique adjustable UC luminescence pattern and directional radiation of peculiar-shaped $NaYF_4$:Yb^{3+}/Er^{3+} microcrystal particle

Qingyan Han, Chengyun Zhang, Chi Wang, Zhaojin Wang, Caixia Li, Wei Gao, Jun Dong, Enjie He, Zhenglong Zhang and Hairong Zheng*

School of Physics and Information Technology, Shaanxi Normal University, 710119, Xi'an, China

*Corresponding author: E-mail: *hrzheng@snnu.edu.cn*.

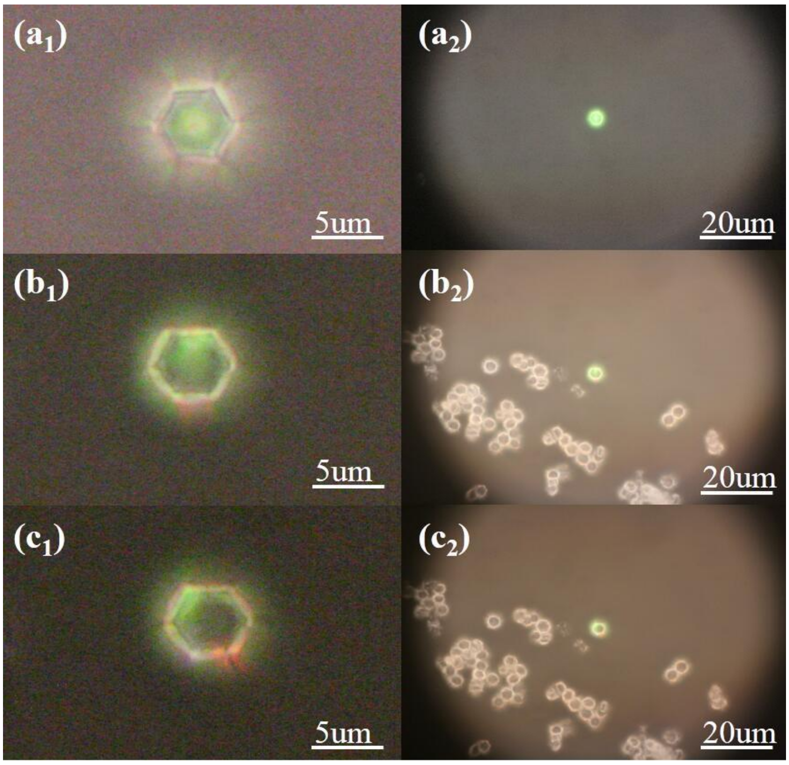

Supporting Information Figure S1 The bright field optical microscope images of single PSβHM. (a_{1-2}), (b_{1-2}), and (c_{1-2}) are luminescence emission spectra when the particle is excited at the center, side, and corner of the microcrystal particle, respectively.

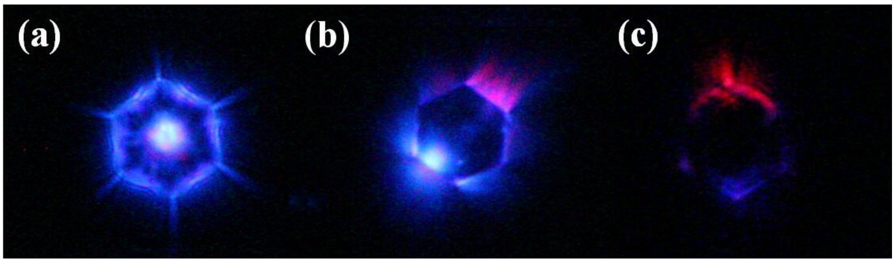

Supporting Information Figure S2 Photographs of upconversion luminescence from the

peculiar-shaped β-$NaYF_4$: 20% Yb^{3+}/ 2% Tm^{3+} hexagonal microcrystal. (a), (b) and (c) are upconversion luminescence emission patterns when the particle is excited at the center, side edge, and corner of the microcrystal particle, respectively.

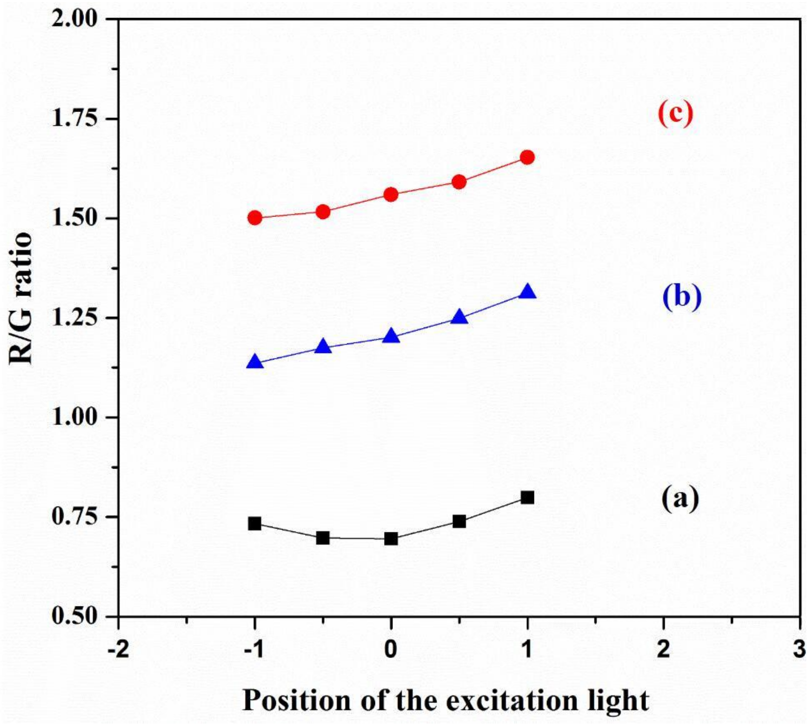

Supporting Information Figure S3 The relationship between the depth of excitation and the R/G ratios of PSβHM. (a), (b), and (c) are luminescence emission spectra when the particle is excited at the center, side, and corner of the microcrystal particle, respectively.

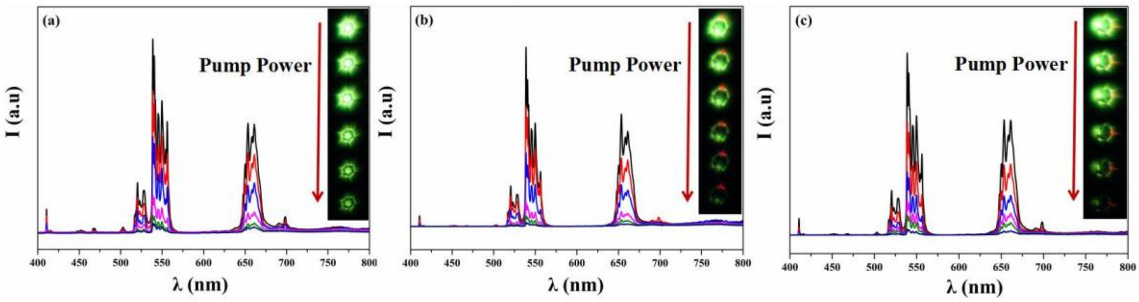

Supporting Information Figure S4 Upconversion emission spectra and luminescent pattern from single PSβHM on a quartz substrate. (a), (b) and (c) are luminescence emission patterns and spectra when the particle is excited at the center, side, and corner of the microcrystal particle under different excitation power, respectively.

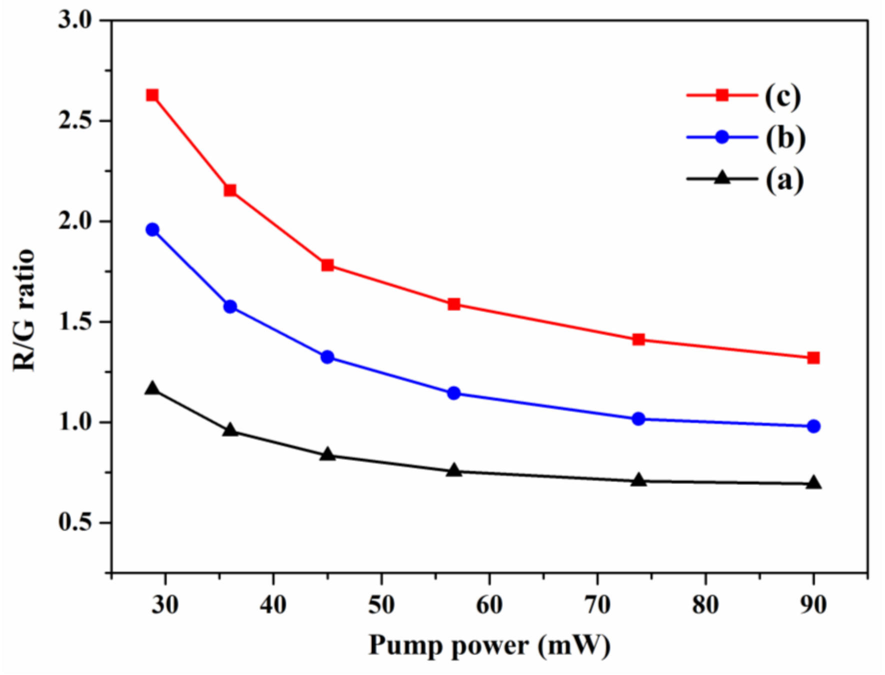

Supporting Information Figure S5 The relationship between the excitation power and the R/G ratios for the three different excitation positions of PSβHM. (a), (b), and (c) are luminescence emission spectra when the particle is excited at the center, side, and corner of the microcrystal particle, respectively.

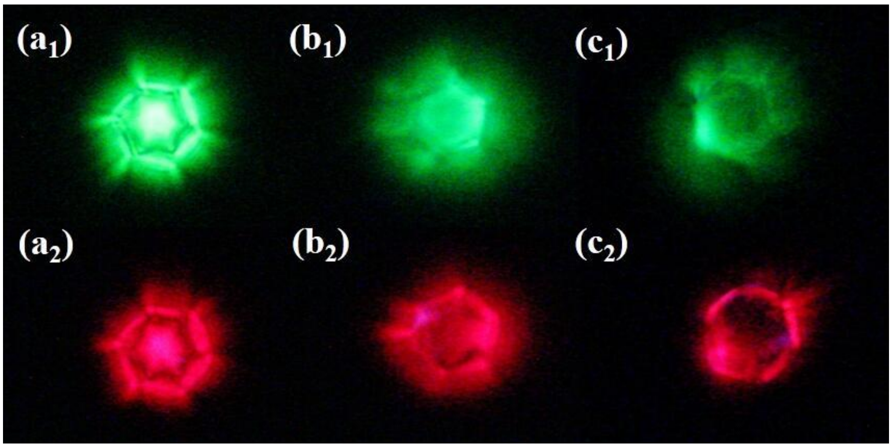

Supporting Information Figure S6 Upconversion emission photos from single PSβHM on a quartz substrate. (a_1), (b_1), and (c_1) are green light emission photos when the particle is excited at side edge and corner of the microcrystal particle, respectively. (a_2), (b_2), and (c_2) are red light emission photos when the particle is excited at side edge and corner of the microcrystal particle, respectively.

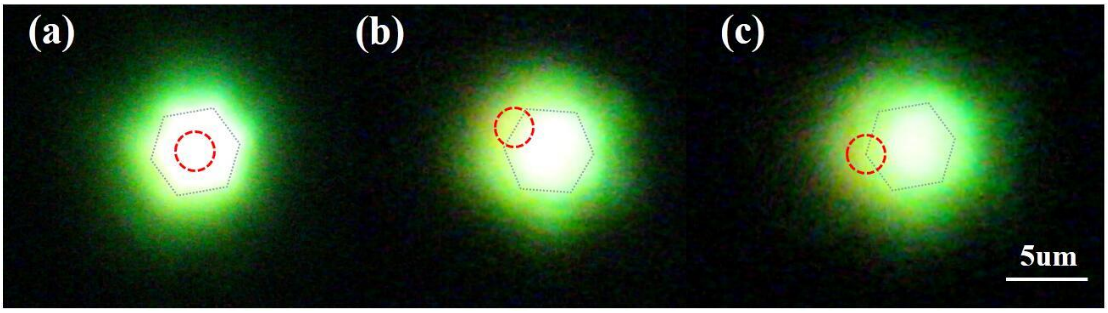

Supporting Information Figure 7 Photographs of upconversion luminescence from the regular hexagonal flat microcrystal plate. (a), (b) and (c) are upconversion luminescence emission patterns when the particle is excited at the center, side edge, and corner of the microcrystal particle, respectively. Red dash-line circles indicate the specific position of the excitation light in the experimental study.
